# Agreement between estimated computed tomography perfusion ischemic core and follow-up infarct on diffusion-weighted imaging

**DOI:** 10.1186/s13244-022-01334-0

**Published:** 2022-12-13

**Authors:** Wenjin Yang, Jan W. Hoving, Miou S. Koopman, Manon L. Tolhuisen, Henk van Voorst, Olvert A. Berkheme, Jonathan M. Coutinho, Ludo F. M. Beenen, Bart J. Emmer

**Affiliations:** 1 Neurovascular Center, Changhai Hospital, Naval Medical University, Shanghai, China; 2 Department of Radiology and Nuclear Medicine, Amsterdam UMC Location University of Amsterdam, Amsterdam, The Netherlands; 3 Department of Biomedical Engineering and Physics, Amsterdam UMC Location University of Amsterdam, Amsterdam, The Netherlands; 4 Department of Neurology, Amsterdam UMC Location University of Amsterdam, Amsterdam, The Netherlands

**Keywords:** Computed tomography perfusion, Diffusion-weighted imaging, Cerebral infarction, Reperfusion

## Abstract

**Background:**

Computed tomography perfusion (CTP) is frequently performed during the diagnostic workup of acute ischemic stroke patients. Yet, ischemic core estimates vary widely between different commercially available software packages. We assessed the volumetric and spatial agreement of the ischemic core on CTP with the follow-up infarct on diffusion-weighted imaging (DWI) using an automated software.

**Methods:**

We included successfully reperfused patients who underwent endovascular treatment (EVT) with CTP and follow-up DWI between November 2017 and September 2020. CTP data were processed with a fully automated software using relative cerebral blood flow (rCBF) < 30% to estimate the ischemic core. The follow-up infarct was segmented on DWI imaging data, which were acquired at approximately 24 h. Ischemic core on CTP was compared with the follow-up infarct lesion on DWI using intraclass correlation coefficient (ICC) and Dice similarity coefficient (Dice).

**Results:**

In 59 patients, the median estimated core volume on CTP was 16 (IQR 8–47) mL. The follow-up infarct volume on DWI was 11 (IQR 6–42) mL. ICC was 0.60 (95% CI 0.33–0.76), indicating moderate volumetric agreement. Median Dice was 0.20 (IQR 0.01–0.35). The median positive predictive value was 0.24 (IQR 0.05–0.57), and the median sensitivity was 0.3 (IQR 0.13–0.47). Severe core overestimation on computed tomography perfusion > 50 mL occurred in 4/59 (7%) of the cases.

**Conclusions:**

In patients with successful reperfusion after EVT, CTP-estimated ischemic core showed moderate volumetric and spatial agreement with the follow-up infarct lesion on DWI, similar to the most used commercially available CTP software packages. Severe ischemic core overestimation was relatively uncommon.

## Key points

- CTP-estimated ischemic core has moderate volumetric and spatial agreement with the infarct lesion on 24 h follow-up DWI.
- The studied CTP software shows similar performance to the most used CTP softwares and can be used interchangeably.
- Severe ischemic core overestimation by the CTP software was relatively uncommon.

## Background

Computed tomography perfusion (CTP) enables quantification of the blood flow through the brain and is increasingly used in the diagnostic workup of acute ischemic stroke [1–3]. Although CTP-estimated ischemic core volume is not recommended to select patients for endovascular treatment (EVT) in the 0–6 h time window, it is an important determinant to determine EVT eligibility in the 6–24 h time window [4, 5]. In several previous studies, CTP-estimated ischemic core volume was used to predict outcome of patients within 6 h after stroke onset for EVT [6–8]. Furthermore, the current AHA/ASA and ESO/ESMINT guidelines recommend to use the mismatch between the CTP-estimated core and penumbral volume determine EVT-eligibility in patients who present beyond 6 h after stroke onset [1, 2].

Various previous studies have assessed the volumetric and spatial agreement of CTP-based ischemic core estimations after successful reperfusion using follow-up diffusion-weighted imaging (DWI) as a reference [4, 9–14]. Commercial software packages from different vendors—using different perfusion parameters and parameter thresholds for core and penumbra estimations—show discrepant results [15]. An increasingly used commercially available CTP analysis software is the perfusion analysis software available in StrokeViewer [16www.nicolab.com/strokeviewer]. The StrokeViewer platform is a CE marked platform which comprises various—FDA-cleared—stroke technology applications including arterial occlusion location detection, collateral status quantification, ASPECTS scoring, hemorrhage segmentation, and analysis of the perfusion status in acute ischemic stroke. Although the diagnostic performance of several applications available on the StrokeViewer platform have been studied and published before [17, 18], the automated perfusion software available in StrokeViewer has not been volumetrically and spatially compared with 24 h FU DWI in a clinical validation set. Given the increased use of the StrokeViewer platform and the fact that treatment decisions may be based on CTP-estimated results—especially in the 6-24 h time window–, it is of great importance to determine the diagnostic performance of the perfusion algorithm available on the StrokeViewer platform and compare this to the performance of other commercially available software packages. Other commonly used software packages for CTP analysis are Rapid CTP (RapidAI, Menlo Park, CA, USA) and syngo.via (Siemens Healthcare, Forchheim, Germany).

We aim to evaluate the volumetric and spatial agreement of CTP-based ischemic core estimations by comparing the results from the StrokeViewer CTP analysis software with follow-up DWI—acquired at a median of approximately 24 h—using a previously validated method and dataset which was created for validation purposes [13, 14].

## Methods

### Study design

We retrospectively included consecutive patients with acute proximal anterior circulation occlusion presented to our comprehensive stroke center who underwent EVT between November 2017 and September 2020. Study inclusion criteria were as follows: patients underwent treatment within 24 h after symptom onset; occlusion of the proximal anterior circulation (internal carotid artery [ICA], M1, or M2 segment of the middle cerebral artery [MCA]); available baseline CTP and follow-up diffusion-weighted imaging (DWI) at approximately 24 h (median) after EVT; successful reperfusion treatment (defined as expanded Treatment in Cerebral Infarction [eTICI] score 2b-3). Patients were excluded if CTP or DWI data could not be processed due to poor acquisition or severe artifacts (e.g., no timely arrival of contrast medium or too little acquisition time points available in the CTP source data). All imaging data were anonymized before the analysis to ensure confidentiality.

### Image acquisition

CTP data were acquired on a dual-source 192-slice scanner (70 kVp, 12 cm coverage; SOMATOM Force, Siemens Healthcare, Forchheim, Germany) or a 128-slice scanner (80 kVp, 10 cm coverage; Siemens SOMATOM Definition AS+, Siemens Healthcare, Forchheim, Germany) depending on availability. Acquired images were reconstructed to 5 mm slices. All scans were performed followed by intravenous injection of 35 mL iodinated non-ionic contrast agent (Iomeron 300, iomeprol, 300 mg iodine/mL; Bracco Imaging Deutschland GmbH, Konstanz, Germany) with an injection rate of 6 mL/s and 2 s delay after contrast injection. The CTP acquisition protocol was as follows: 25 scans were acquired at 1.5 s apart, followed by the acquisition of 6 scans at 3 s apart, resulting in a total of 31 scans over a period of approximately 56 s. Follow-up DWI (*b* = 0 s/mm^2^ and *b* = 1000 s/mm^2^) and apparent diffusion coefficient (ADC) images were acquired on either a 1.5 T scanner (MAGNETOM Avanto fit, Siemens Healthcare, Erlangen, Germany) or a 3.0 T scanner (Ingenia 3.0 T, Philips Healthcare, Best, the Netherlands) with a slice thickness of 5 mm.

### CTP data post-processing

CTP data were processed using the fully automated perfusion analysis software available on the StrokeViewer platform. The data processing involved automated registration, segmentation, and motion correction. Additional information on patient motion (correction) and contrast bolus curves were automatically provided by the software. The ischemic core was defined as brain tissue with a relative cerebral blood flow (rCBF) < 30% compared with the contralateral hemisphere, while hypoperfused brain tissue was defined as a Tmax > 6 s. Summary maps were generated depicting ischemic core and hypoperfusion with corresponding volumes. All CTP results were visually checked by two trained observers (W.Y., J.W.H.).

### Follow-up imaging assessment

The DWI follow-up data were co-registered to baseline CTP images using Elastix [19]. The methods of this co-registration approach have been published before [13, 14]. Follow-up infarct lesion was assessed on DWI images (median 23 h) using a previously published semi-automated segmentation method using a Deepmedic network [20, 21]. All segmentations were visually checked by an expert neuroradiologist (> 20 years of experience) who was blinded to all clinical information except for the occlusion side [21]. Apparent diffusion coefficient (ADC) maps were consulted to determine if T2 shine-through lesions were suspected to be present on DWI data—using the following threshold: ADC ≤ 620 × 10^−6^ mm^2^/s [22]. If necessary, the semi-automated segmentation results were manually adjusted using ITK-SNAP. We decided to use DWI for follow-up infarct assessment since DWI is widely used as an accurate assessment tool for infarct core and considered to be more sensitive for (sub)acute ischemic lesions compared to fluid-attenuated inversed recovery (FLAIR) [23, 24].

### Data analysis

The volume difference between the estimated CTP core volume and DWI lesion was calculated as the lesion volume on DWI minus the CTP-estimated ischemic core volume. Negative volumes indicated overestimation by CTP or ‘ghost infarct core’ [25].

The volumetric agreement between the CTP-estimated ischemic core and the lesion on follow-up DWI imaging was evaluated using the intraclass correlation coefficient (ICC) and 95% confidence intervals. We chose a two-way mixed model, absolute agreement type, and average measures to calculate ICC (the degree of agreement is interpreted with ICC < 0.5 = poor agreement, ICC 0.5–0.75 = moderate agreement, ICC 0.75–0.9 = good agreement, and ICC > 0.9 = excellent agreement) [26]. We performed Bland–Altman analyses to present the volumetric agreement. When there was a proportional bias assessed by linear regression, a logarithmic transformation of the data would be performed to meet the requirements of standard Bland–Altman [27].

To quantify the spatial agreement between ischemic core volume on the CTP and final ischemic volume on the DWI, we calculated the Dice similarity coefficient using FSLMaths [28]. The precision (i.e., the positive predictive value) was determined using EvaluateSegmentation [29]. Finally, a sensitivity analysis was performed comparing patients with complete reperfusion to patients with successful, but incomplete reperfusion (i.e., eTICI 3 vs. eTICI 2b-2c). Spatial agreement statistics from other software packages reported in the literature were compared with the performance of the StrokeViewer CTP software. Descriptive statistics are presented as the median (interquartile range [IQR]) for non-normally distributed continuous variables. Categorical variables are represented as percentages. All statistical analyses were performed with R (R Core Team (2020). R: A language and environment for statistical computing, R Foundation for Statistical Computing, Vienna, Austria. https://www.R-project.org).

## Results

**Fig. 1:**
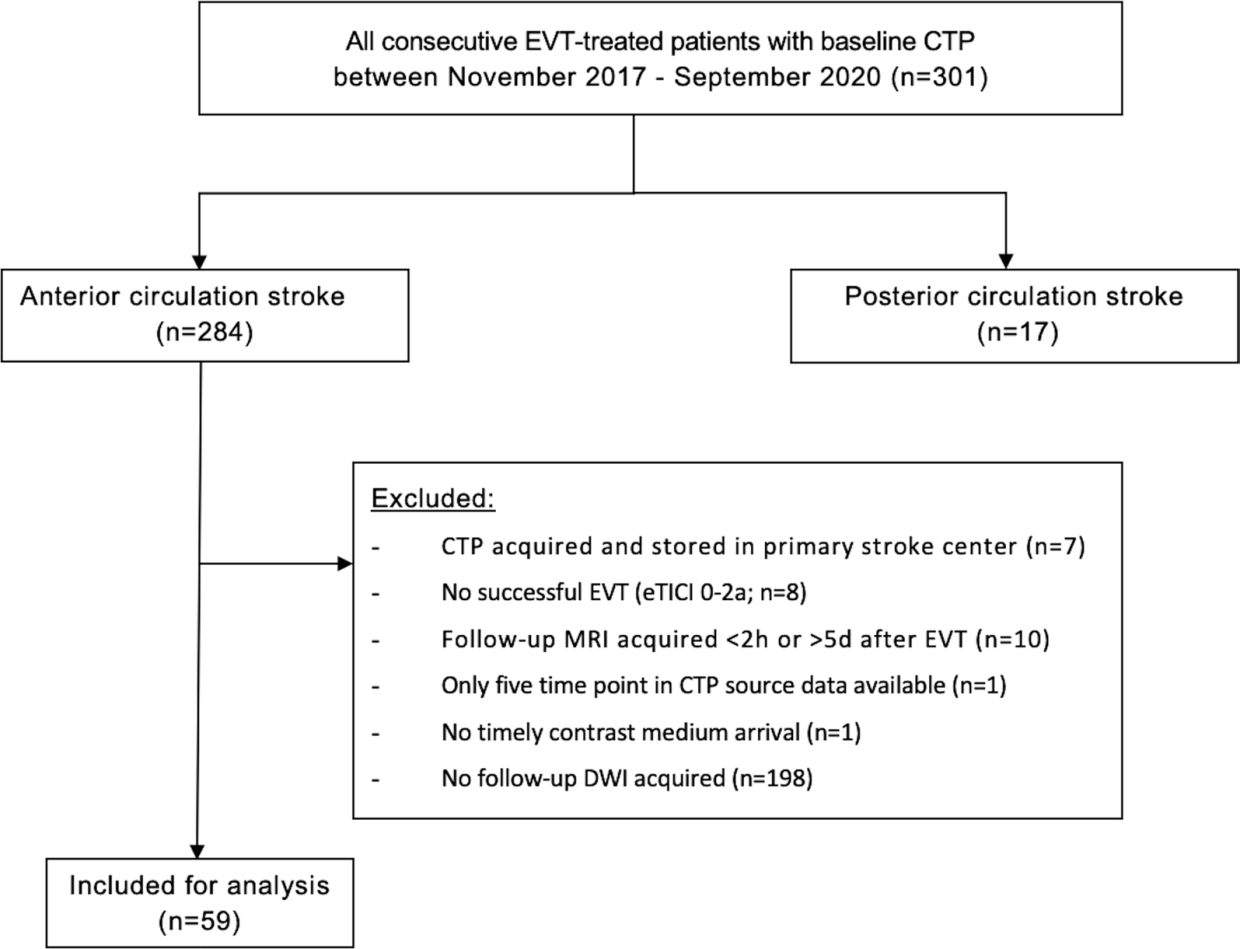
Flowchart of patient selection

Two hundred eighty-four stroke patients with an anterior circulation proximal vessel occlusion at baseline computed tomography angiography (CTA) were presented at our stroke center from November 2017 to September 2020. Eighty-four patients (30%) underwent follow-up DWI imaging. The median time interval from baseline CTP to follow-up DWI was 23 (IQR 18–34) hours. The total range of time interval between CTP and follow-up DWI was 1–113 h. In total, 59 patients were included for analysis (Fig. 1). Forty-nine of 59 (83%) included patients were included in one of the trials from the CONTRAST consortium (i.e., MR CLEAN-NO IV, MR CLEAN-MED or MR CLEAN-LATE) and received follow-up imaging as part of the pre-specified follow-up imaging protocol of the concerning trial [30].

The baseline characteristics of the included patients are presented in Table 1. The median age was 71 (IQR 57–77) years. Most patients (59%) were female. The median National Institutes of Health Stroke Scale Score (NIHSS) was 15 (IQR 9–18), and the median Alberta Stroke Program Early CT Score (ASPECTS) was 8 (IQR 7–10). Intravenous alteplase was administered in 48%. The median time from baseline imaging to reperfusion was 83 (IQR 62–115) minutes.

**Table 1:** Baseline clinical characteristics

	Study cohort (*n* = 59)
*Clinical characteristics*	
Age (yr)–median (IQR)	71 (57–77)
Female–*n* (%)	35 (59)
NIHSS score–median (IQR) [known in]	15 (9–18) [*n* = 55]
ASPECTS–median (IQR) [known in]	8 (7–10) [*n* = 27]
IVT administered–*n* (%)	28 (47)
Onset-to-imaging time (min)–median (IQR) [known in]	83 (58–189) [*n* = 57]
Imaging-to-reperfusion time (min)–median (IQR) [known in]	83 (62–115) [*n* = 58]
Onset-to-groin time (min)–median (IQR) [known in]	140 (103–233) [*n* = 49]
*Imaging characteristics*	
Occlusion location on baseline CTA–*n* (%)	
Intracranial ICA	1 (2)
ICA-bifurcation	9 (15)
M1	41 (70)
M2	2 (3)
Tandem	6 (10)
Collateral status–*n* (%) [known in]	[*n* = 21]
0	2 (3)
1	7 (12)
2	6 (10)
3	6 (10)
Median baseline ischemic core volume on CTP (mL);–median (IQR)	16 (8–47)
*Posttreatment recanalization rate (eTICI)*	
2b	19 (32)
2c	10 (17)
3	30 (51)
Follow-up DWI infarct volume (mL)–median (IQR)	11 (6–42)
Percentage of CTP-estimated ischemic core in DWI lesion (%)–median (IQR)	75 (31–218)
Median time between baseline CTP and follow-up DWI (hours)–median (IQR)	23 (18–34)

*ASPECTS*  Alberta Stroke Program Early CT Score, *CTA*  CT angiography, *CTP*  CT perfusion, *DWI*  diffusion-weighted imaging, *eTICI*  expanded Treatment in Cerebral Infarction, *ICA* = internal carotid artery, *IQR*  interquartile range, *IVT*  intravenous alteplase, *NIHSS*  National Institutes of Health Stroke Scale. If the [known in] number is not shown, the variable was known in all patients

The median volume of baseline ischemic core on CTP and follow-up infarct on DWI was 16.0 (IQR 8.0–47.0) and 10.8 (IQR 5.5–42.2) mL, respectively. The StrokeViewer software indicated that for 4 (7%) patients, some degree of curve truncation was present and results should be interpreted with caution. In addition, for 6 (10%) patients, patient motion was present. Metal artifacts were present in one patient. Data from two (3%) patients could not be processed due to no timely contrast medium arrival and only five available acquisition time points, respectively (Fig. 1).

**Fig. 2:**
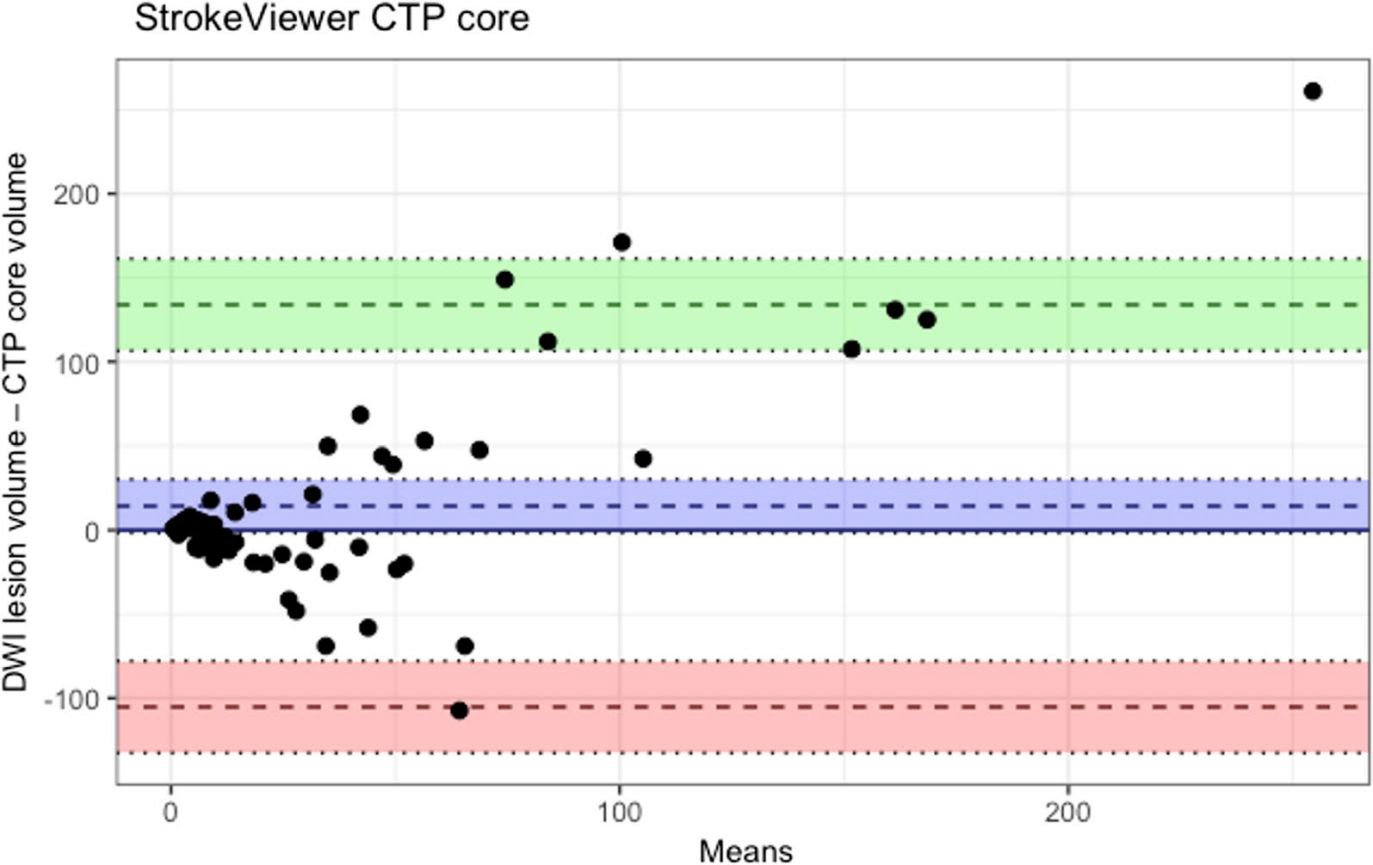
Bland–Altman plot comparing the estimated CTP ischemic core volume and DWI follow-up infarct volume. The mean bias (blue), lower (red) and upper (green) limits of agreement are shown with 95% confidence intervals. The bias with 95% confidence intervals is shown in blue. Negative values indicate overestimation by CTP. *CTP*  CT perfusion, *DWI*  diffusion weighted imaging

The ICC was 0.60 (95% CI 0.33–0.76), indicating moderate volumetric agreement. The median volumetric difference was 16.3 (IQR 5.8–48.2) mL (Table 2). The volume differences showed a non-normal distribution and unequal variance with a fixed bias testified by linear regression. CTP core overestimation > 50 mL occurred in 4/59 (7%) of cases (Fig. 2). Median Dice similarity coefficient was 0.20 (IQR 0.01–0.35). The median positive predictive value was 0.24 (IQR 0.05–0.57) and the median sensitivity was 0.3 (IQR 0.13–0.47) (Table 2).

**Table 2:** Volumetric and spatial accuracy between baseline CTP ischemic lesions and follow-up DWI lesions for patients with eTICI 2b-2c vs. eTICI 3

	eTICI 2b-2c (*n* = 29)	eTICI 3 (*n* = 30)	Total (eTICI 2b-3 [*n* = 59])
Median baseline ischemic core volume on CTP (mL)–median (IQR)	13.0 (5.5–32.5)	20.0 (7.8–47.3)	16.0 (8.0–47.0)
Follow-up DWI infarct volume (mL)–median (IQR)	17.7 (5.6–69.0)	10.1 (5.1–37.3)	10.8 (5.5–42.2)
Median volumetric difference (mL)–median (IQR)	6 (−10 to 47) 17 (7.2–68.7)	−3 (−19 to 5) 13.3 (3.7–43.8)	0 (−12; 21) 16.3 (5.8–48.2)
Median Dice similarity coefficient–median (IQR)	0.12 (0.00–0.35)	0.20 (0.03–0.36)	0.20 (0.01–0.35)
Median positive predictive value–median (IQR)	0.34 (0.12–0.44)	0.27 (0.12–0.52)	0.30 (0.13–0.47)

*CTP*  CT perfusion, *DWI*  diffusion-weighted imaging, *eTICI*  expanded Treatment in Cerebral Infarction, *IQR*  interquartile range

In a sensitivity analysis for patients with complete reperfusion (eTICI 3), median volumetric differences were 13.3 (3.7–43.8) mL. Median ICC was 0.57 (IQR 0.09–0.80) and median Dice similarity coefficient was 0.20 (IQR 0.03–0.36), respectively. Median positive predictive value was 0.21 (0.40–0.45) and median sensitivity was 0.27 (0.12–0.52) (Table 2).

## Discussion

Our study showed moderate volumetric and spatial agreement between ischemic core estimated by StrokeViewer and the follow-up infarct lesion on the DWI for patients with acute proximal anterior circulation vessel occlusion and successful reperfusion. Severe ischemic core overestimation > 50 mL by CTP was rare. The value of evaluation on ischemic core with StrokeViewer was comparable with other commercial software packages [13, 15, 31, 32].

The spatial agreement between ischemic core estimated by StrokeViewer and follow-up infarct on DWI (Dice 0.20) was comparable with the previously reported agreement between CTP-estimated ischemic core and follow-up infarct on DWI for RapidAI CTP (Dice 0.24) and syngo.via (Dice 0.16–0.21, depending on which core estimation approach was used) [14]. However, it should be noted that the agreement of Rapid CTP was assessed using a different dataset, which complicates direct comparison to our findings [13]. Since we used a similar co-registration approach as previously published studies assessing the diagnostic performance of other CTP software package, we aimed to limit any bias which could be introduced by using different co-registration methods [13, 14].

Furthermore, the spatial agreement between the CTP ischemic core estimated by the StrokeViewer perfusion analysis software and the follow-up DWI lesion was limited. Possible explanations for the limited spatial agreement include infarct growth during the imaging-to-reperfusion interval and during the period between baseline imaging and follow-up imaging—particularly in patients without complete reperfusion. In addition, echoplanar image distortion, slice thicknesses difference, and suboptimal registration of thick slice images could have affected the spatial agreement.

The assessment of ischemic core based on baseline imaging is of great value for clinical outcome prediction of patients with acute ischemic stroke [5, 8, 33, 34] The ischemic core volume estimated by CTP impacts clinical outcome and efficacy of thrombectomy for acute ischemic stroke patients [31, 35, 36]. CTP is widely used in emergency settings because of its widespread availability and relatively short acquisition times compared to MRI. Previous studies showed that CBF corresponded better with the follow-up DWI lesion than other parameters such as cerebral blood volume, time to peak, mean transit time [37–40]. A threshold of rCBF < 31% was considered as the optimal threshold in identifying infarct core [37]. However, different postprocessing algorithms from different software vendors could alter the optimal thresholds. RapidAI CTP software, using rCBF < 30% for ischemic core, could assess ischemic core volume more accurately than other commercial software [33]. StrokeViewer CTP software—using a similar rCBF threshold of < 30% yielded similar moderate spatial and volumetric agreement with follow-up DWI compared to RapidAI software [13].

DWI was used as a reference standard because of its high sensitivity for infarcted brain tissue, which has been widely accepted in research and clinical practice [12, 24, 41]. Previous studies compared CTP-estimated ischemic core to follow-up DWI using either contemporaneous imaging (i.e., CTP and DWI acquired before treatment and within a short interval between baseline and follow-up imaging) or compared baseline CTP to follow-up –post-treatment—DWI imaging comparison [13, 33, 37, 38, 42]. In the current era of endovascular treatment, it is not feasible to acquire contemporaneous baseline CTP and DWI, as this would delay treatment. The timing between baseline and follow-up imaging is an important factor to consider when performing volumetric accuracy studies as the infarct is likely to expand in the time interval between baseline and follow-up imaging. Particularly in patients with incomplete reperfusion after EVT (e.g., eTICI 2b) [43]. We tried to limit the effect of these limitations, but it is likely that infarct growth affected both the volumetric and spatial agreement. Additionally, potential effect of infarct growth during the short interval from baseline imaging to reperfusion remained existent, even though a previous study did not find that spatial and volumetric accuracy was reduced in patients within a short imaging-to-reperfusion time interval [13].

Strengths of this study include the use of a single acquisition protocol, which reduced the heterogeneity of the CTP source data. Moreover, we assessed the follow-up infarct volume on DWI at a median of approximately 24 h after reperfusion, which minimizes the influence of infarct volume inflation due to brain edema [43]. Furthermore, DWI is considered the most sensitive and accurate imaging to delineate the follow-up infarct [44].

Several limitations to this study should be noted: First, this was a retrospective study only including patients with follow-up DWI. In the Dutch healthcare system, follow-up imaging (either DWI or CT) is only performed for research purposes in most centers and therefore only sporadically performed in routine clinical practice. In our dataset, the vast majority of patients were included in either the MR CLEAN-NO IV (ISRCTN80619088), the MR CLEAN-MED (ISRCTN76741621), or the MR CLEAN-LATE (ISRCTN19922220) trial. All included patients underwent follow-up imaging as part of the standard follow-up imaging protocol of the concerning trial, which were all part of the Collaboration for New Treatments of Acute Stroke (CONTRAST) consortium [30]. Second, we did not compare different thresholds to assess an optimal threshold for StrokeViewer. Third, although the interval from CTP to reperfusion was relatively short and all subjects had successful reperfusion, there was still a potential underestimation of ischemic core volume on CTP due to infarct growth, especially for patients with incomplete reperfusion (i.e., of eTICI 2b). Fourth, we did not evaluate the reproducibility and reliability of StrokeViewer in this study with reference to other software packages. Fifth, co-registration between two different imaging modalities is not optimal and might negatively affect the results of our spatial agreement analysis. Finally, all CTP data in this study were acquired on two scanners developed by a single vendor, hence our results might not be generalizable to different scanners and scanners from different vendors. Last, most patients included in this study presented within 6 h from the onset and our results might not be generalizable to patients with longer onset-to-imaging intervals. Although CTP is currently not recommended for EVT selection for these patients, it might still be a valuable diagnostic imaging tool in the acute setting with useful implications for outcome prediction of acute ischemic stroke patients.

## Conclusions

In patients with successful reperfusion after EVT, CTP ischemic core estimated by Nicolab StrokeViewer showed moderate volumetric and spatial agreement with the follow-up infarct on diffusion-weighted imaging, similar to the most used commercially available CTP software packages. Severe ischemic core overestimation was rare.

## Data Availability

Individual patient data cannot be made available under Dutch law as we did not obtain patient approval for sharing individual, coded patient data. In line with privacy regulations, publication of individual patient data as well as syntax files and output of statistical analyses is forbidden by the Data Privacy Officer of the Amsterdam UMC. All syntax files and output of statistical analyses are available on reasonable request to the corresponding author.

## Abbreviations

ADC: Apparent diffusion coefficient
ASPECTS: Alberta stroke program early CT score
CTA: Computed tomography angiography
CTP: Computed tomography perfusion
Dice: Dice similarity coefficient
DWI: Diffusion-weighted imaging
eTICI: Expanded treatment in cerebral infarction
EVT: Endovascular treatment
FLAIR: Fluid-attenuated inversed recovery
ICA: Internal carotid artery
ICC: Intraclass correlation coefficient
MCA: Middle cerebral artery
NIHSS: National Institutes of Health Stroke Scale Score
